# *Trypanosoma brucei gambiense* Infections in Mice Lead to Tropism to the Reproductive Organs, and Horizontal and Vertical Transmission

**DOI:** 10.1371/journal.pntd.0004350

**Published:** 2016-01-06

**Authors:** Nicolas Biteau, Corinne Asencio, Julien Izotte, Benoit Rousseau, Muriel Fèvre, Davita Pillay, Théo Baltz

**Affiliations:** 1 Microbiologie Fondamentale et Pathogénicité, UMR 5234, Centre National de la Recherche Scientifique (CNRS), Université de Bordeaux, Bordeaux, France; 2 Animalerie A2, Université de Bordeaux, Bordeaux, France; New York University School of Medicine, UNITED STATES

## Abstract

*Trypanosoma brucei gambiense*, transmitted by the tsetse fly, is the main causative agent of Human African trypanosomosis in West Africa and poses a significant health risk to 70 million people. Disease progression varies depending on host immunity, but usually begins with a haemo-lymphatic phase, followed by parasite invasion of the central nervous system.

In the current study, the tropism of *T*. *b*. *gambiense* 1135, causing a low level chronic ‘silent’ infection, was monitored in a murine model using bioluminescence imaging and PCR. A tropism to the reproductive organs, in addition to the central nervous system, after 12–18 months of infection was observed. Bioluminescent analysis of healthy females crossed with infected males showed that 50%, 62.5% and 37.5% of the female mice were subsequently positive for parasites in their ovaries, uteri and brain respectively. Although PCR confirmed the presence of parasites in the uterus of one of these mice, the blood of all mice was negative by PCR and LAMP. Subsequently, bioluminescent imaging of the offspring of infected female mice crossed with healthy males indicated parasites were present in the reproductive organs of both male (80%) and female (60%) offspring.

These findings imply that transmission of *T*. *b*. *gambiense* 1135 occurs horizontally, most probably via sexual contact, and vertically in a murine model, which raises the possibility of a similar transmission in humans. This has wide reaching implications. Firstly, the observations made in this study are likely to be valid for wild animals acting as a reservoir for *T*. *b*. *gambiense*. Also, the reproductive organs may act as a refuge for parasites during drug treatment in a similar manner to the central nervous system. This could leave patients at risk of a relapse, ultimately allowing them to act as a reservoir for subsequent transmission by tsetse and possibly, horizontally and vertically.

## Author Summary

Human African trypanosomosis (HAT) caused by *Trypanosoma brucei gambiense* is a serious disease threatening 70 million people in West Africa. The parasite is transmitted by the tsetse fly, and initially multiplies in the bloodstream of the mammalian host, before progressing to the central nervous system. Using a strain of *T*. *b*. *gambiense* transfected with a gene for luminescent detection that causes a chronic infection with very low parasitaemia, we found that the parasite is capable of entering the reproductive organs of both male and female mice. Subsequently, crossing infected male mice with healthy females resulted in some female mice becoming infected. Furthermore, female mice infected directly with *T*. *b*. *gambiense* parasites and crossed with healthy males, produced offspring which were also shown to be positive for parasites. These experiments demonstrated that *T*. *b*. *gambiense* 1135 is transmitted both horizontally, most probably by sexual contact, and vertically in mice. If these alternate modes of transmission are analogous to the situation in humans, this has drastic implications for future control measures of HAT as parasites may avoid the immune system and treatment by accumulating in the reproductive organs as well as the CNS.

## Introduction

Human African trypanosomosis (HAT) is caused by the protozoan parasite *Trypanosoma brucei gambiense* in West and Central Africa and *T*. *b*. *rhodesiense* in East Africa. *T*. *b*. *gambiense* is responsible for more than 90% of all reported cases and results in a chronic infection whereas *T*. *b*. *rhodesiense* usually causes a more acute disease [1,2,3]. In endemic countries, 70 million people over 1.55 million km^2^ are at risk of HAT infection [4]. Furthermore, from 2000–2010, 94 travellers from 19 different non-endemic countries were diagnosed with HAT [5,6].

*T*. *b*. *gambiense* HAT is characterised by an early haemo-lymphatic stage (stage 1) which can occur 1–2 weeks after the initial infection and can persist for several months to years [7] which is then followed by a meningo-encephalitic late stage (stage 2) where parasites invade the central nervous system (CNS) [8,9]. However, it has recently been established that HAT may lead to various disease states, including a latent state characterised by a chronic, asymptomatic stage 1 and no progression to stage 2, or asymptomatic stage 2 [10,11]. Such variation in disease progression is likely to be affected by host genetics [10,12,13].

A tentative diagnosis of HAT is based on clinical symptoms and serological screening using the card agglutination test (CATT). This is followed by detection of parasites in blood, lymph or cerebrospinal fluid using several techniques including microscopic and molecular methods, primarily PCR of the 18S ribosomal RNA gene [14,15]. Most recently, rapid diagnostic tests have been evaluated for HAT [16], and it has been established that the use of two rapid tests in combination has excellent specificity and is very promising as a new method of field diagnosis [17]. However, the foremost difficulty in HAT diagnosis stems from the very low and fluctuating parasite load during chronic infection [9,12,18,19]. In addition, treatment failure is difficult to detect since neither PCR nor antibody detection from blood samples is sufficiently sensitive, though PCR of cerebrospinal fluid may identify some positives [20].

Tsetse fly transmission of *T*. *b*. *gambiense* is largely regarded as the primary mode of transmission of HAT and, unlike *T*. *b*. *rhodesiense*, *T*. *b*. *gambiense* is generally transmitted from human to human [21,22]. Attempts to verify the presence of an animal reservoir for *T*. *b*. *gambiense* have been inconclusive due to the limitations of the detection methods [22,23,24,25]. However, a study of transmission cycles of HAT in Cameroon concluded that treatment of human cases alone would not eliminate the disease due to the probably re-introduction via an animal reservoir [26]. Interestingly, there have been a few reported cases of vertical transmission of HAT [27]. Although WHO guidelines acknowledge that mother-to-child transmission via the placenta is a possibility, the frequency at which it occurs is unknown. On the other hand, reports of the sexual transmission of HAT is close to non-existent, with only one possible case documented [28].

A previous study showed that the typical latent disease state associated with *T*. *b*. *gambiense* HAT could be reproduced in mice. Specifically, strain 1135 of *T*. *b*. *gambiense* produced an infection characterised by very low parasitaemia and CNS involvement [29]. In the present study *ex vivo* bioluminescent imaging (BLI) was used to monitor the organ tropism of *T*. *b*. *gambiense* strain 1135 during experimental infection in mice. Due to the very low levels of parasitaemia during infection, it was also necessary to develop a sensitive nested PCR to confirm the presence of parasites. The frequency of horizontal transmission was investigated by crossing *T*. *b*. *gambiense* infected male mice with healthy females. The extent of vertical transmission was examined by monitoring the progress of disease in the offspring of infected female mice using BLI and PCR.

## Methods

### Ethics statement

All mice procedures were carried out in strict accordance with the French legislation (Rural Code articles L 214–1 to L 214–122 and associated penal consequences) and European Union (Directive 2010/63/EU Protection of Animals Used for Scientific Purposes) guidelines for the care of laboratory animals and were approved by the Ethical Committee of Centre National de la Recherche Scientifique, Région Aquitaine and by the University of Bordeaux animal care and use committee. All efforts were made to minimize animal suffering.

### Parasite strains and mouse infections

*T*. *b*. *gambiense* 1135 was originally isolated from a patient in Dalao, Ivory Coast in 1991, and subsequently transfected with the Rluc-pHD309 plasmid as described [29] to generate strain 1135 (Rluc). BALB/c J mice were purchased from Charles River (L'Arbresle, France) and used for experiments at five to six weeks of age. Post-infection, male mice were always housed individually, while females were housed as groups of five, at a maximum. Female (n = 16) and male (n = 18) mice were infected i.p. with 1–5 × 10^6^ with *T*. *b*. *gambiense* 1135 (RLuc) parasites diluted in PSG (phosphate buffered saline with 10% glucose: 137 mM NaCl, 10 mM phosphate, 2.7 mM KCl, pH 7.4) and the clinical signs monitored over 12–18 months. Subsequently, eight of the female mice and six male mice were sacrificed for *ex vivo* BLI analysis of various organs.

To investigate horizontal transmission, male mice (n = 5) infected with strain 1135 (Rluc) for three months were then crossed with healthy female mice (n = 8) for a maximum period of two months of co-housing. During this period of co-housing, one male mouse was co-housed with either one or two females. Blood was taken from the female mice approximately 3.5, 5 and 6 months post crossing for PCR. The female mice were sacrificed approximately 7 months post crossing for BLI and PCR of various organs. In order to rule out the possibility of transmission occurring via an mechanism (other than sexually), one infected female was co-housed with three non-infected females for a period of three months. After this period, *in vivo* BLI was conducted and showed that all three females remained non-infected.

Similarly for analysis of vertical transmission, female mice (n = 10) were infected with strain 1135 (Rluc) and crossed with healthy male mice (n = 5). Five male and five female offspring from five different litters were subsequently sacrificed for *ex vivo* BLI of the organs at 2–3 months of age. In addition, one pregnant female mouse was sacrificed for PCR of its organs as well as two of the embryos.

### Bioluminescence imaging

For *ex vivo* bioluminescence imaging, mice were sacrificed and organs removed and soaked for 5 minutes in coelenterazine (Promega) (20 μg/ml PBS) prepared as previously described [29]. Light emission was recorded immediately in real time with a Biospace Imaging System (Biospace lab, Paris, France), and the result corresponded to the values measured when the signal was optimal and stable for at least 3 minutes. Quantification of BLI signals was performed on selected regions of interest (ROI). For the comparison of signals of the same organ between different mice, the signal was integrated for 30 seconds, the area of the ROI kept constant and the intensity given as photon/second/steradian/cm^2^ (ph/s/sr/cm^2^). Maximum and minimum values were fixed respectively at 2 × 10^4^ and 6 × 10^3^ ph/s/sr/cm^2^, smoothing = 3. A ratio of the signal intensity of the sample tissue against the mean signal intensity of at least two non-infected controls was calculated. A sample was considered positive when this ratio was above 1.5 unless the signal intensity was below the mean signal intensity plus standard deviation of the controls, in which case it was considered negative. Note that while individual organs were compared to the corresponding organ of a non-infected control to determine a cut-off, photographs taken were normalised as entire pictures, thus not normalised for each individual organ.

### DNA extraction from blood

In order to concentrate trypanosomes, enhance the sensitivity of trypanosome detection and remove polymerase enzyme inhibitors, red blood cells (RBCs) were lysed prior to DNA extraction using a commercial kit (QIAamp DNA mini kit, Qiagen). Blood samples (150 μl) were collected in 100 μl PSG and 30 μl heparin (30 mg/ml in 150 mM NaCl), mixed with 2250 μl of 1 × RBC lysis buffer (155 mM NH_4_Cl, 10 mM KHCO_3_, 0.1 mM EDTA pH 8), incubated 5 min at 20°C and centrifuged (700 g, 15 min, 20°C). The supernatant was carefully discarded, the pellet resuspended in 200 μl PBS and the DNA extracted using the QIAamp kit. After extraction, DNA was eluted in 50 μl sterile water and quantified by spectrophotometry (LVis-plate, FLUOstar Omega, BMG Labtech).

### DNA extraction from tissues

Organs of sacrificed mice were collected in 2 ml sterile tubes and immediately frozen on dry ice. Only one ovary and one testis were collected and only part (25–150 mg) of the brain, kidney, liver, lung, uterus, spinal cord, and spleen were collected. Depending on the weight, tissues were mechanically disrupted with 200–600 μl of PBS with a 0.1 ml Potter-Elvehjem. The homogenate was then treated with an equal volume of buffer ATL (Qiagen) and 10% (v/v) of proteinase K (Qiagen). The samples were mixed by vortex and incubated at 56°C for 12 h with occasionally mixing during incubation. After centrifugation (10 min, 14 000 *g*), samples were transferred in a 1.5 or 15 ml Phase Lock Gel tube (PLG light, 5 Prime) depending on the volume. Two DNA extractions with equal volumes of sample and phenol:chloroform:isoamyl alcohol (25:24:1) and chloroform:isoamyl alcohol (24:1) were performed. An ethanol precipitation was then performed on the aqueous phase [1:10 volume of 3 M NaAc pH 5.2, 2.5 (v/v) 100% ethanol] 2 h at 20°C, centrifuged (30 min, 4°C, maximum speed) and the pellet was washed twice with 70% ethanol. To remove the last traces of ethanol, a quick spin and aspiration of any residual fluid was performed. Depending on the observed precipitates, the pellets were air dried and resuspended 12 to 24 h at 60°C in 50–300 μl of TER (10 mM Tris, 1 mM EDTA, 0.1 mg/ml RNAase-A) with repeated mixing of the samples. The DNA samples were quantified by spectrophotometer.

### PCR and LAMP detection of trypanosomes

In this study, two Trypanozoon-specific PCR methods were used to detect the presence of parasite DNA. The first was the TBR-PCR [30] previously described to be highly sensitive for HAT diagnosis [29,31,32]. TBR1/2 primers were used to amplify a 164 bp highly repeated sequence of mini-chromosome satellite DNA. In order to get more specific PCR products, the forward primer was modified as described in S1 Table.

Concurrently, a second PCR was adapted from the same region amplified by the pMUTec F/R primers (S1 Table). These PCR primers were developed to amplify a repetitive sequence (237 bp) of *T*. *evansi* [33]. The corresponding PCR product belongs to the ingi retrotransposon composed of a 4.7 kb fragment bordered by two separate halves of the RIME retroelement [34]. PCR primers were customized to develop a specific and sensitive nested PCR for the detection of very low parasitaemia. After the first amplification step with pMUTec-F8/TBingi-R1 in 96 PCR sealed plates, 1 μl was taken for second nested PCR step with TBingi-F1/pMUTec-R2. To avoid contamination, DNA extractions and PCR were performed in separate rooms and filter tips used for all pipetting steps.

All PCR were carried out in a final volume of 20 μl containing 0.8 μM of each primer, 0.2 mM dNTP, 1× incubation buffer with 2.5 mM MgCl2, 1.5 unit of GoTaq polymerase (Promega), 1 μl of tissue-extracted or 2 μl blood-extracted DNA. PCR conditions with TBR 1N/2 were performed as described [29].

Briefly, PCR conditions with pMUTec/TBingi primers were as follow: an initial denaturation step of 5 min at 95°C; five cycles of touch down amplification with 10 s denaturation at 95°C, 15 s hybridization at 62°C (-1°C at each subsequent step) and either 40 s or 30 s elongation at 72°C for pMUTec-F8/TBingi-R1 and TBingi-F1/pMUTec-R2 primer sets respectively; thirty cycles of amplification with 10 s denaturation at 95°C, 15 s hybridization at 57°C and 40 s or 30 s elongation at 72°C for pMUTec-F8/TBingi-R1 and TBingi-F1/pMUTec-R2 primers sets respectively; a final extension step of 5 min at 72°C.

PCR amplification was performed in duplicate in three different assays. Purified *T*. *b*. *gambiense* DNA was used as positive control and two negative controls with *T*. *congolense* DNA and without DNA were performed. Amplicons (10 μl) were visualised by UV on 1.8% agarose gel, stained with 0.5 mg/ml ethidium bromide. Results were considered positive when the correct size products were observed.

To detect the parasite DNA, the RIME-LAMP assay was also used (Loopamp *Trypanosoma brucei* Detection Kit, Eiken Chemical CO. LTD., Japan) based on species-specific primers targeting the repetitive insertion mobile element (RIME) of the Trypanozoon subgenus group [35]. The test was performed according to the manufacturer instructions. Briefly, the tubes containing the dried reagents were filled with 25 μl containing 2 μl of blood DNA extracts or 0.5–1 μg of tissue extracts. Negative and positive controls were performed for each test and detection of amplified products was done under UV light.

## Results

### Parasite tropism to reproductive organs

**Fig 1:**
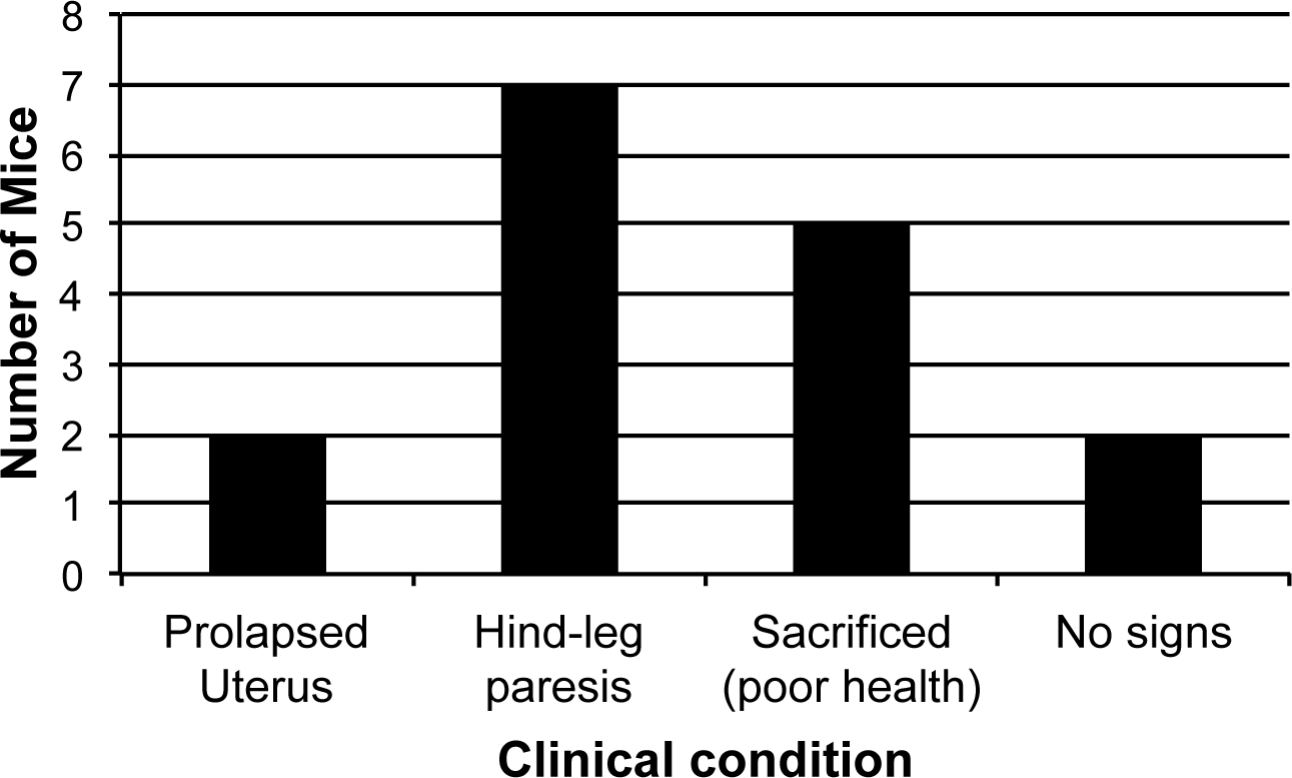
Clinical signs observed in female mice (n = 16) following 12–18 months of infection with *T*. *b*. *gambiense* 1135 (Rluc). Mice were infected i.p. with 1–5 × 10^6^ *T*. *b*. *gambiense* 1135 (Rluc) parasites and clinical signs monitored over 12–18 months.

**Fig 2:**
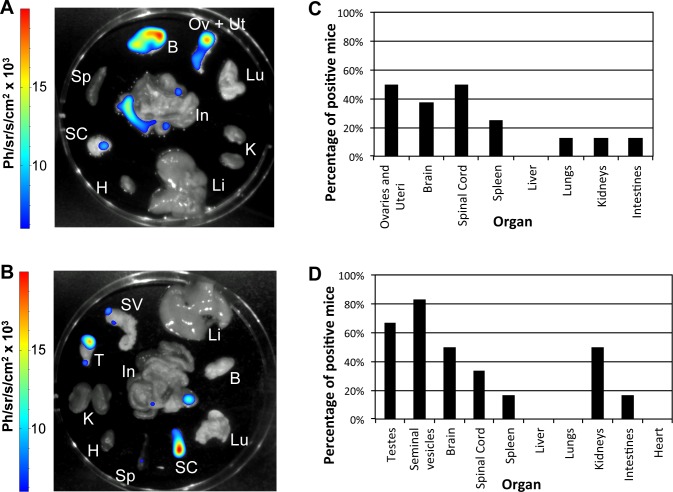
Tropism of *T*. *b*. *gambiense* 1135 (Rluc) in male (n = 6) and female (n = 8) mice infected for 12–18 months analysed by bioluminescence. Representative BLI of *ex vivo* organs of a single infected (A) female (mouse 23797g, see S2 Table) and (B) male (mouse 27711SM, see S2 Table) mouse. The colour scale to the right of the image indicates the colour intensity in ph/sr/cm^2^/s. Ov, ovaries; Ut, uterus; T, testes; SV, seminal vesicles; B, brain; SC, spinal cord; Sp, spleen; Li, liver; Lu, lungs; K, kidneys; In, intestines; H, heart. Percentage of positive (C) female (n = 8) and (D) male mice (n = 6) per organ by BLI.

Female (n = 16) and male (n = 18) mice were infected with *T*. *b*. *gambiense* strain 1135 (Rluc) and their health monitored for up to 18 months post infection. During this period, 2/16 of the female mice suffered genital prolapse and 7/16 displayed hind-leg paresis (Fig 1). In addition, one mouse showed a significant splenomegaly. In the case of male mice, clinical signs observed up to 12 months included inflammation of the testes (4/18) and hind-leg paresis (9/18). Also, three male mice had severe splenomegaly. *Ex vivo* BLI analysis (female n = 8, male n = 6) of the organs (Fig 2) showed the presence of parasites not only in the CNS (brain and spinal cord), but also in the reproductive organs of a minimum of 50% of the infected male and female mice. In both male and female infected mice, the number of mice positive by BLI for parasites in the reproductive organs was comparable to, or greater than, the number of mice which were positive for parasites in the CNS. For example, five male mice had a positive BLI signal in their seminal vesicles, and four of these five had a similar positive signal in their testes. However, only three mice had a positive BLI signal in the brain and only 1/4 male mice with hind-leg paresis had a positive BLI for the spinal cord. The BLI signal of the reproductive organs, spinal cord and brain of individual infected female and male mice can be found in S1 Fig. Similarly, the fold increase of BLI signal compared to the control is available in S2 Table for both infected female and male mice. Interestingly, the average magnitude of signal in the reproductive organs was higher than that in the brain.

### Horizontal transmission

**Fig 3:**
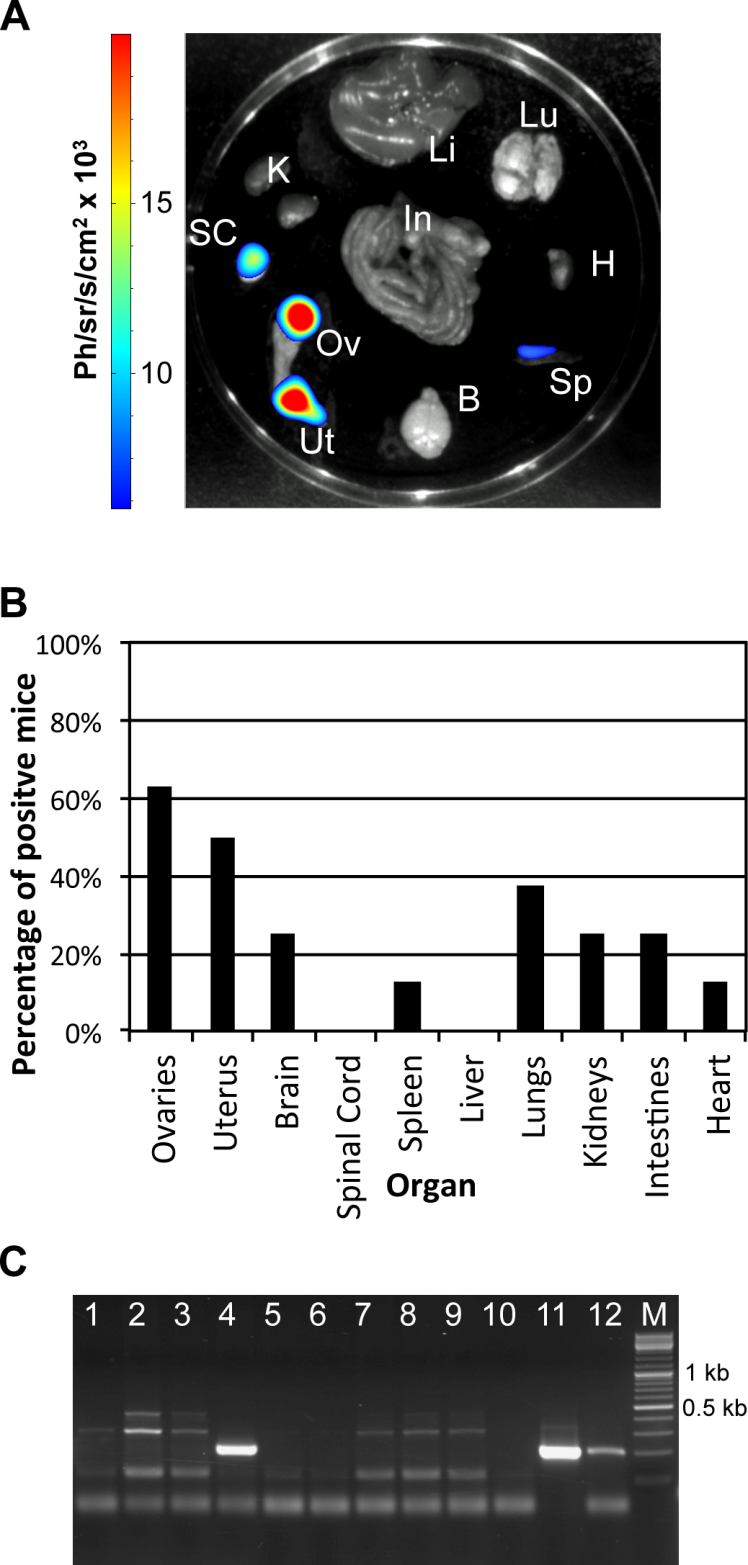
Investigation of the horizontal transmission of *T*. *b*. *gambiense* 1135 (Rluc) by crossing healthy female (n = 8) mice with infected male mice (n = 5). BLI signal from *ex vivo* organs of female mice (n = 8) crossed with *T*. *b*. *gambiense* 1135 infected males (n = 5) examined 7 months after crossing. A. BLI of *ex vivo* organs of a representative female mouse (mouse 229, see S3 Table). Ov, ovaries; Ut, uterus; B, brain; SC, spinal cord; Sp, spleen; Li, liver; Lu, lungs; K, kidneys; In, intestines; H, heart. The colour scale to the right of the image indicates the colour intensity in ph/sr/cm^2^/s. B. Percentage of positive female mice per organ. C. 1.8% agarose gel of PCR using pMUTec/TBingi nested primers on organs of female mice 229 and 228. Lane 1: spinal cord negative control; lane 2: spleen negative control; lane 3: ovary mouse 229; lane 4: uterus mouse 229 (band 201 bp); lane 5: spinal cord negative control; lane 6 spinal cord mouse 229; lane 7: spleen mouse 229; lane 8: ovary mouse 228; lane 9: uterus mouse 228; lane 10: spinal cord mouse 228; lane 11: genomic DNA *T*. *b*. *brucei* (nested PCR of the first PCR positive control); lane 12: genomic DNA *T*. *b*. *brucei* (nested PCR positive control); lane M: GeneRuler DNA ladder (ThermoScientific).

The tropism of the parasite to the reproductive organs raises the question of horizontal transmission, especially via sexual contact. To this end, crosses were conducted between five *T*. *b*. *gambiense* 1135 (Rluc) infected male mice and eight healthy females. PCR using two different primer sets and LAMP was conducted on the blood of the female mice taken 3.5, 5 and 6 months post-crossing and both techniques proved to be negative at each point (S4 Fig). BLI analysis of the *ex vivo* organs of a representative female mouse, as well as the number of mice with positive organs are shown (Fig 3). Seven of the eight female mice were all positive for at least one organ by BLI. Only one of the eight female mice was not positive by BLI nor PCR (mouse 275, S3 Table). All four female mice that were positive for parasites by BLI in the uterus, were also positive by BLI in the ovaries. PCR with the pMUTec/TBingi nested primers indicated that a uterine DNA sample of one animal (mouse 229, S3 Table) was positive (Fig 3C), in addition to being the strongest positive signal by BLI. Note that the initial amplification of the uterine-tissue DNA extracted from this mouse with the pMUTec/TBingi primers was not visible by agarose gel, and only the second amplification with the nested primers produced a visible product. This indicated that the parasite load in the organs of these animals is at the detection limit of this technique, and that this PCR is indeed very specific for the target sequence. Surprisingly, BLI analysis showed that only two of the female mice were positive for parasites in the brain, and none were positive for parasites in the spinal cord (S3 Table). The BLI signals of the uteri and ovaries of the individual female mice can be found in S2 Fig.

### Vertical transmission

**Fig 4:**
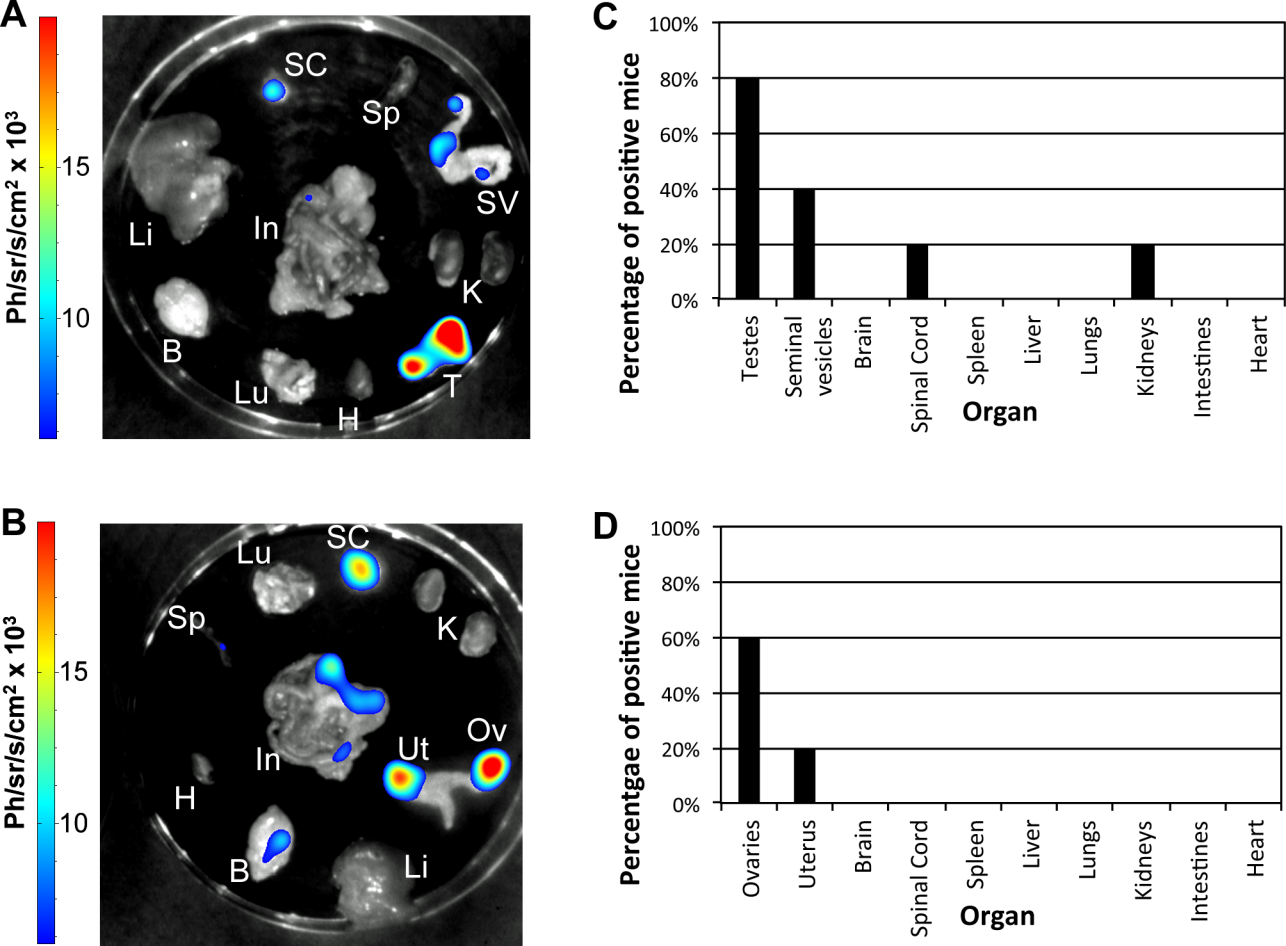
Analysis of vertical transmission by BLI of *ex vivo* organs of offspring (2–3 months old) of *T*. *b*. *gambiense* 1135 (Rluc) infected females (n = 10) crossed with healthy males (n = 5). BLI of *ex vivo* organs of a representative single (A) male (mouse 32486(1), see S4 Table) and (B) female (mouse 32925–1, see S4 Table) offspring. Ov, ovaries; Ut, uterus; T, testes; SV, seminal vesicles; B, brain; SC, spinal cord; Sp, spleen; Li, liver; Lu, lungs; K, kidneys; In, intestines; H, heart. The colour scale to the right of the image indicates the colour intensity in ph/sr/cm^2^/s. Percentage of (C) male and (D) female mice positive by BLI per organ.

In order to study the extent of vertical transmission, ten *T*. *b*. *gambiense* 1135 (Rluc) infected female mice were crossed with five healthy male mice. PCR using the pMUTec/TBingi nested primers conducted on one pregnant female mouse indicated that the uterus, placenta, spleen and spinal cord were all positive (S5 Fig). More importantly, nested PCR indicated that two of the embryos were also positive for parasites (S5 Fig). Subsequently, from the resulting offspring, five of each sex were chosen and sacrificed for *ex vivo* organ BLI analysis two to three months after birth (Fig 4). BLI analysis indicated that four out of five male offspring and three out of five female offspring had parasites in their reproductive organs. Interestingly, only one male (mouse 32486(2), S4 Table) and none of the female offspring gave a positive BLI signal for the CNS. Overall, the frequency of vertical transmission can be estimated at 60–80% since from the ten offspring examined, only three (one male and two female) were not positive for any organ by BLI. However, it is likely that this is an underestimate due to the limits of sensitivity of both the BLI and the nested PCR. The BLI signals from individual male and female offspring can be found in S4 Fig.

## Discussion

In a previous study, recombinant *T*. *b*. *gambiense* parasites containing a R-Luc transgene were used to follow the distribution of parasites in the host and to monitor the progression of the disease [29]. This model was used to show that the parasite exhibited a tropism not only to the CNS, but also to the spleen and lungs of the host within the first three months of infection. In the current study, extended periods of infections result in parasites moving towards the reproductive organs, thus raising the possibility of horizontal (notably via sexual contact) and vertical transmission. It should be noted that the strain of *T*. *b*. *gambiense* used in this study (1135) was defined as being a silent type of infected, i.e. displaying a very low parasitaemia. This model, with extremely low parasitaemia, renders transmission via contaminated blood as highly unlikely, but is similarly, not suited to immunohistochemical detection since the background associated with this technique requires that only intact whole labelled parasites can be counted as positive. BLI provides a more sensitive level of detection, especially when parasites are present as single detached cells and not in foci. BLI coupled with *in situ* imaging of transgenic parasites expressing a fluorescent protein may overcome this issue. In addition, perfusion of the animals and the rinsing of organs prior to BLI were intended to eliminate the possibility of contamination of tissue samples with blood.

Initial examination of parasite tropism showed that mice infected with *T*. *b*. gambiense 1135 (Rluc) for an extended period developed genital prolapse and hind-leg paresis among other clinical signs. Although hind-leg paresis has previously been associated with CNS infection in *T*. *evansi* [36], genital prolapse has been observed after prolonged infection with *T*. *equiperdum* and is due to infection of the reproductive organs themselves [37]. It has previously been established that *T*. *b*. *gambiense* and *T*. *b*. *brucei* accumulate in the testes of rabbits, voles, and other laboratory rodents during the course of infection [38,39]. *T*. *vivax*, a causative agent of trypanosomosis in animals, has also been shown to localise to the ovaries of infected goats [40]. Furthermore, *T*. *b*. *gambiense* infection in humans has been associated with various reproductive disorders including amenorrhoea, infertility, spontaneous abortions and impotence [8,41], which have previously been attributed to parasite invasion of the CNS [42]. In addition, a single case of orchitis as a symptom of HAT has been reported [43]. In light of the present study, it is necessary to examine whether the presence of parasites in the reproductive organs themselves contributes to these pathologies.

Although trypanosome tropism to the testes was initially thought to be associated with an affinity for lower temperatures, a study showed that mice with induced cryptorchidism still accumulated parasites in the testes [38]. Furthermore, the current study demonstrated the presence of parasites in both the uterus and ovaries of infected females as established by BLI. Thus, temperature is unlikely to be a factor in trypanosome tropism to the reproductive organs.

Relapse of *T*. *b*. *gambiense* infection in humans has been reported as varying from 5 to 50% after treatment with the stage 2 drug, melarsoprol [44,45]. This large variation in the reported relapse rate is likely to be due to the difficulty in diagnosing treatment failures by either PCR of blood or antibody detection methods [20]. Currently, the most likely explanations for the high relapse rate were the existence of drug resistant-parasites or host factors influencing metabolism of the drug [45]. However, the reproductive tissue tropism demonstrated in the current study provides an alternative explanation since parasites may be protected from drug circulating in the vascular system [39], in a similar manner to those parasites which cross the blood-brain barrier and multiply in the CNS [46].

There is a precedent for sexual transmission of trypanosomes since *T*. *b*. *equiperdum*, the tissue parasite also belonging to the Trypanozoon sub-genus, is transmitted primarily during coitus, causing the disease dourine in equines [47]. However, there has only been one documented case of possible sexual transmission of HAT in humans to date [28]. Therefore, this mode of transmission has been considered either extremely rare or non-existent [1]. In the current study, the strain of *T*. *b*. *gambiense* used produced a very low parasite load which proved to be at the detection limit of both the PCR developed in this study and the BLI using the RLuc gene. Although it was not possible to examine the semen of infected mice for parasites due to experimental constraints, PCR and BLI did show the presence of parasites in the seminal vesicles. These findings, together with the very low parasitaemia making other haematogenous routes unlikely, and absence of transmission by co-housing infected and non-infected females, point towards sexual transmission as the most likely route of horizontal transmission. Ultimately, the frequency of horizontal transmission observed in this study is likely to be an underestimation due to the limits of the sensitivity of the two techniques, BLI and PCR, used for detection. However, it is evident that this mode of transmission does occurs in mice, and thus may occur more frequently than expected in humans in late infection.

Vertical transmission of the animal pathogenic trypanosomes *T*. *vivax* and *T*. *congolense* has previously been observed, although the frequency at which this occurs is unknown [48,49,50]. *T*. *equiperdum* is also known to be infrequently transmitted from mother to offspring, but it is not clear if transmission occurs during pregnancy or through suckling from the infected mother [51]. It is well established that *T*. *cruzi*, the parasite causing Chagas disease in humans in South America, is transmitted vertically with approximately 13 835 cases a year [52,53]. However, there could be multiple mechanisms of transmission of *T*. *cruzi* due to the possibility of the parasite being intracellular, which is not the case for African trypanosomes. In the current study, the offspring of infected female mice also appeared to be infected, although the parasites seemed to be limited to the reproductive organs and the central nervous system. It is possible that parasite undergoes metabolic changes when in the tissues, adapting them to the lower oxygen environment of the tissues. This alternate form could subsequently be restricted to these organs and unable to re-colonise the vascular system due to the higher concentration of oxygen. However, as the offspring were only observed three months after birth, the parasite could eventually disseminate to other major organs. It remains to be investigated if the parasite can effectively be transmitted by tsetse following vertical transmission.

Vertical transmission of HAT has previously been defined as the positive diagnosis of a newborn of an infected mother within the first five days of life and, to date, only 17 cases of vertical HAT infection have been documented since 1933 [27]. It is, therefore, not surprising that vertical transmission has been considered to be infrequent [54]. However, there are several reasons why the five day diagnosis definition of vertical transmission may be too narrow [27], the most persuasive of which is the difficulty of diagnosis of HAT [14]. Another contribution to the low number of vertically transmitted cases to date is the fact that HAT causes sterility and abortion; therefore, few infected women give birth. For these reasons, together with the data presented in the current study, it is likely that the frequency of vertical transmission of HAT in humans has been underestimated to date and this has serious implications in terms of the control of the disease since only pentamidine, a first stage drug, is safe for use by pregnant women [20].

The current study represents the first demonstration of horizontal (probably via sexual contact) and vertical transmission of *T*. *b*. *gambiense* in a mouse model, and has wide reaching implications for the control of HAT. Thorough research needs to be done on the tropism of the parasite to reproductive organ tissues since this would have repercussions for the development of new drugs that would need to be able to penetrate the blood-tissue barrier (brain and testes) in order to clear parasites from the affected tissues. Furthermore, the possibility of sexual transmission of HAT should be considered given that control of the tsetse vector is currently the main focus of disease prevention, and since transmission not requiring the vector could have global epidemiological consequences. [55]. Also, it is evident that vertical transmission of HAT may occur at much higher frequency than historically supposed, making it imperative that protocols are established to monitor and treat infected pregnant women. The vertical transmission of *T*. *b*. *gambiense* demonstrated in the current study implies that it may be possible for a silent reservoir of parasites to exist, especially since the disease can progress differently in different hosts. Also, it should be considered that silent infections resulting in horizontal and vertical transmission may also occur in other Trypanozoon parasites. Finally, it is essential that the viability of the parasites in the tsetse vector be evaluated after horizontal and vertical transmission.

## Supporting Information

S1 Fig BLI signal from *ex vivo* organs of individual female (n = 8) and male mice (n = 6) infected with *T*. *b*. *gambiense* 1135 for 12–18 months. (A) Ovaries and uterus, (B) brain, and (C) spinal cord of female mice. (D) Testes, (E) seminal vesicles, and (F) brain of male mice.
(DOCX)

S2 Fig BLI signal from *ex vivo* organs of individual females (n = 8) crossed with *T*. *b*. *gambiense* 1135 infected males examined seven months post-crossing. (A) Ovaries. (B) Uterus.
(DOCX)

S3 Fig BLI signal from *ex vivo* organs of male (n = 5) and female (n = 5) offspring (2–3 months old) of *T*. *b*. *gambiense* 1135 infected females (n = 10) crossed with healthy males (n = 5). BLI signal of the (A) testes (B) seminal vesicles of individual male offspring. BLI signal of the (C) uterii and (D) ovaries of individual female offspring.
(DOCX)

S4 Fig Investigation of the horizontal transmission of *T*. *b*. *gambiense* 1135 (Rluc). 1.8% agarose gel of PCR using Tbingi-F1/pMUTec-R2 nested primers on the blood of 8 female mice taken 3.5, 5 and 6 months post-crossing with *T*. *b*. *gambiense* 1135(Rluc) infected male. PCR has been done in duplicate for the three series and for the two blood extracts controls. Respectively lane 1 to 11: 1-nested PCR of the first PCR negative control (water); 2-nested PCR of the first PCR positive control (1 ng *T*. *b*. *gambiense* DNA); 3-nested PCR of the first PCR negative control (1 ng *T*. *congolense* DNA); 4-negative control (water) of the nested PCR; 5-positive control (1 ng *T*. *b*. *gambiense* DNA) of the nested PCR; 6-negative control (1 ng *T*. *congolense* DNA) of the nested PCR; 7-healty mouse blood extract; 8-T. b. gambiense 1135 mouse blood extract; 9-negative control (water) of the first PCR; 10-positive control (1 ng *T*. *b*. *gambiense* DNA) of the first PCR; 11-negative control (1 ng *T*. *congolense* DNA) of the first PCR. Lane M: GeneRuler DNA ladder (Thermo Scientific).
(DOCX)

S5 Fig Investigation of the vertical transmission of *T*. *b*. *gambiense* 1135 (Rluc). 1.8% agarose gel of PCR using Tbingi-F1/pMUTec-R2 nested primers on organs of an infected *T*. *b*. *gambiense* 1135 pregnant female mouse. Respectively lane 1 to 19: 1-lung; 2-intestine; 3-liver; 4-kidney; 5-placenta; 6-spleen; 7-brain; 8-ovary; 9-spinal cord; 10-uterus; 11-heart; 12-embryo; 13-embryo; 14-healthy mouse blood extract; 15-nested PCR of the first PCR negative control (water); 16-nested PCR of the first PCR positive control (1 ng *T*. *b*. *gambiense* DNA); 17-nested PCR of the first PCR negative control (1 ng *T*. *congolense* DNA); 18-negative control (water) of the nested PCR; 19-positive control (1 ng *T*. *b*. *gambiense* DNA) of the nested PCR. Lane S: other tissue samples. Lane M: GeneRuler DNA ladder (Thermo Scientific).
(DOCX)

S1 Table Primer sequences used in the study for the sensitive and specific detection of very low parasitaemia. (DOCX)

S2 Table Fold increase of BLI signal to control from *ex-vivo* organs of *T*. *b*. *gambiense* 1135 infected male (n = 6) and female mice (n = 8) 12–18 months post-infection. Positive samples are indicated in bold^a^.
(DOCX)

S3 Table Investigation of horizontal transmission of *T*. *b*. *gambiense* 1135 from infected male mice (n = 5) to healthy female mice (n = 8) examined by *ex-vivo* organ BLI and PCR seven months post-crossing. Positive samples are indicated in bold^a^.
(DOCX)

S4 Table Fold increase of BLI signal to control for five female and five male offspring of *T*. *b*. *gambiense* 1135 infected females (n = 10) and healthy male mice (n = 5) examined at 2–3 months of age. Positive values+ are indicated in bold ^a^.
(DOCX)
