# Supplementary material for: Trypanosoma brucei gambiense Infections in Mice Lead to Tropism to the Reproductive Organs, and Horizontal and Vertical Transmission
Source: PLoS Negl Trop Dis. 2016 Jan 6;10(1):e0004350. doi: 10.1371/journal.pntd.0004350 (PMC4703293; doi:10.1371/journal.pntd.0004350)
Supplement: S1 Fig — (A) Ovaries and uterus, (B) brain, and (C) spinal cord of female mice. (D) Testes, (E) seminal vesicles, and (F) brain of male mice. (DOCX) [file pntd.0004350.s001.docx]

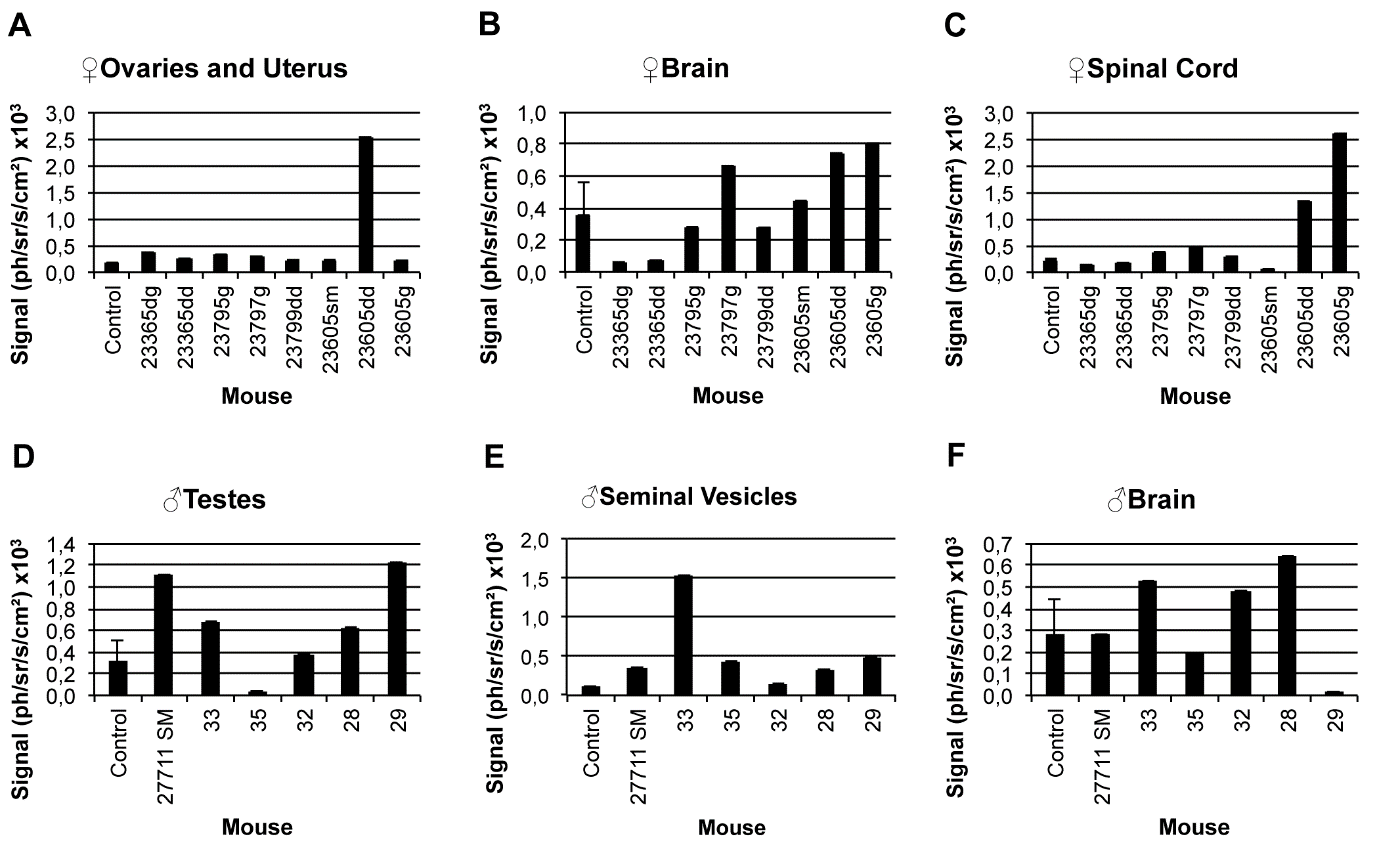


S1 Figure. BLI signal from *ex vivo* organs of individual female (n=8) and male mice (n=6) infected with *T. b. gambiense* 1135 for 12-18 months. (A) Ovaries and uterus, (B) brain, and (C) spinal cord of female mice. (D) Testes, (E) seminal vesicles, and (F) brain of male mice.
