# Supplementary material for: Trypanosoma brucei gambiense Infections in Mice Lead to Tropism to the Reproductive Organs, and Horizontal and Vertical Transmission
Source: PLoS Negl Trop Dis. 2016 Jan 6;10(1):e0004350. doi: 10.1371/journal.pntd.0004350 (PMC4703293; doi:10.1371/journal.pntd.0004350)
Supplement: S2 Fig — (A) Ovaries. (B) Uterus. (DOCX) [file pntd.0004350.s002.docx]

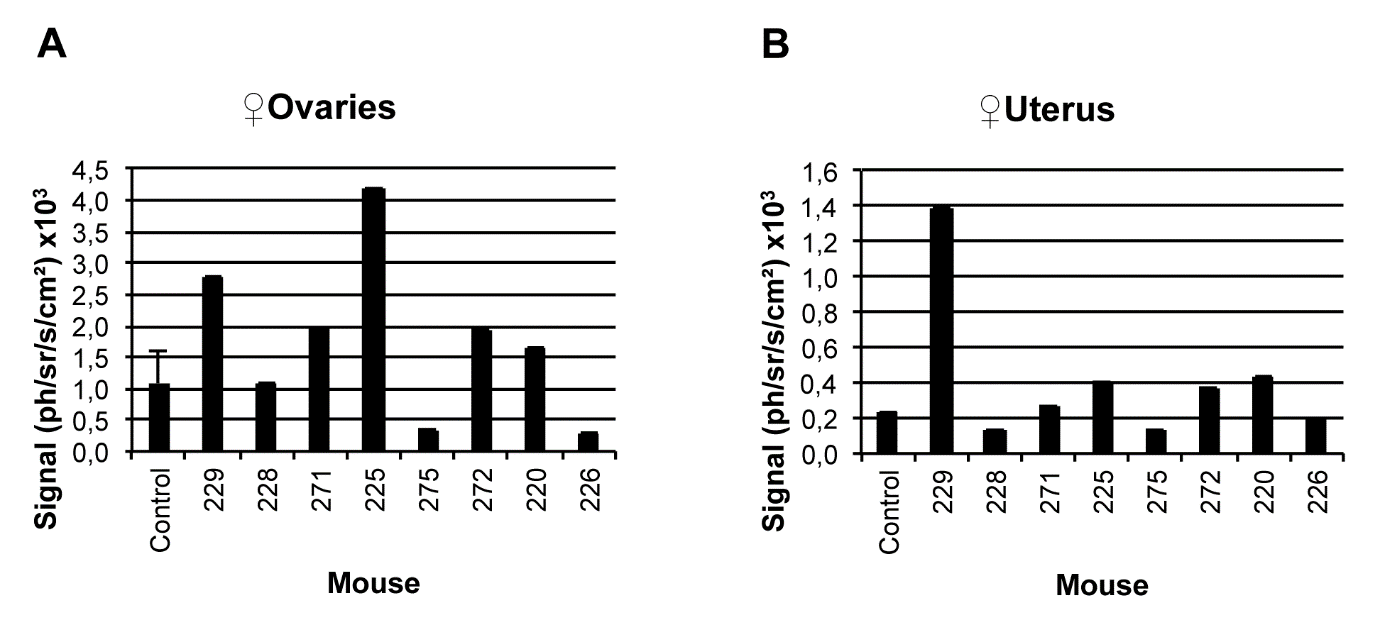


S2 Figure. BLI signal from *ex vivo* organs of individual females (n=8) crossed with *T. b. gambiense* 1135 infected males. (A) Ovaries. (B) Uterus.
