# Supplementary material for: Trypanosoma brucei gambiense Infections in Mice Lead to Tropism to the Reproductive Organs, and Horizontal and Vertical Transmission
Source: PLoS Negl Trop Dis. 2016 Jan 6;10(1):e0004350. doi: 10.1371/journal.pntd.0004350 (PMC4703293; doi:10.1371/journal.pntd.0004350)
Supplement: S3 Fig — BLI signal of the (A) testes (B) seminal vesicles of individual male offspring. BLI signal of the (C) uterii and (D) ovaries of individual female offspring. (DOCX) [file pntd.0004350.s003.docx]

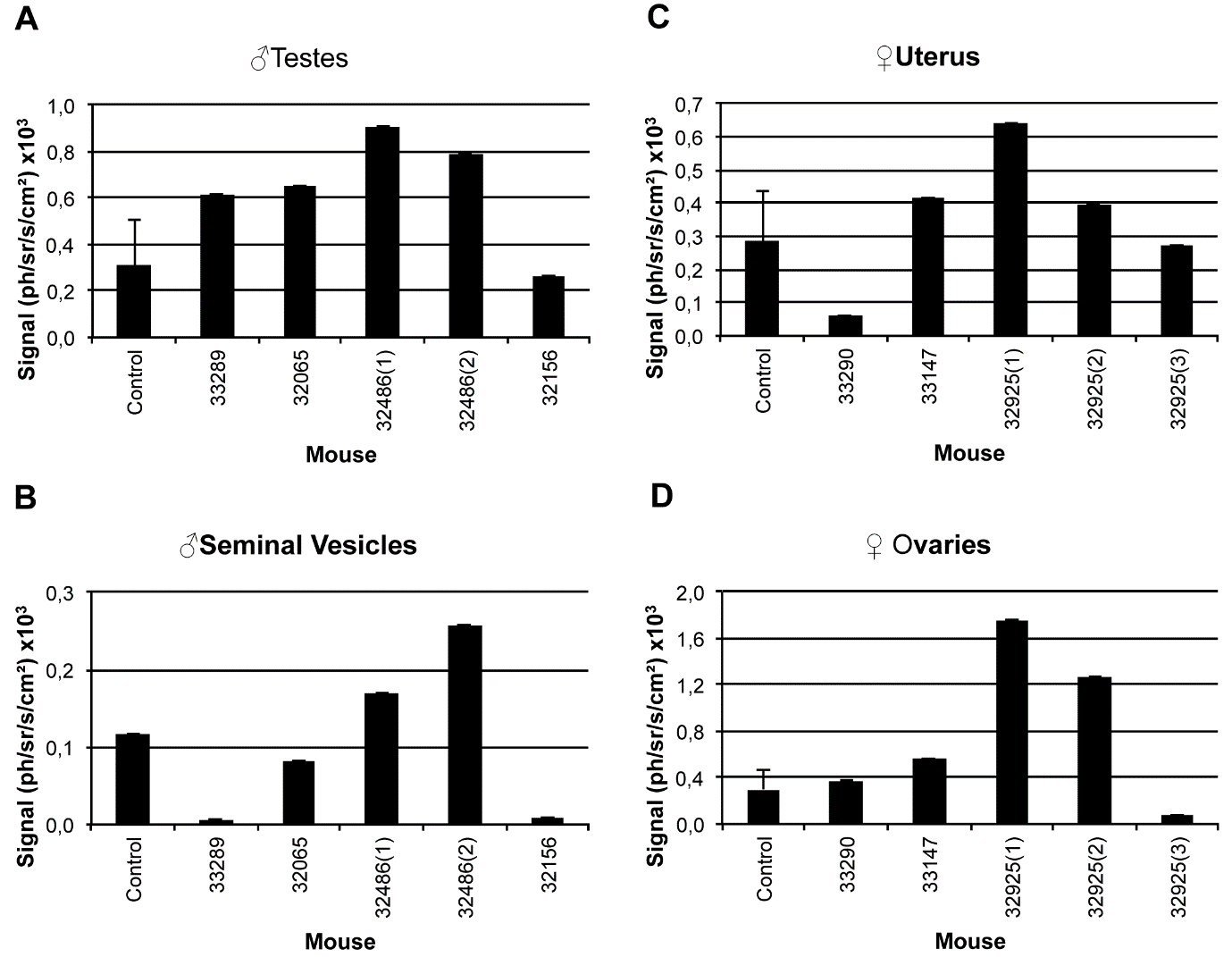


S3 Figure. BLI signal from ex vivo organs of male (n=5) and female (n=5) offspring of *T. b. gambiense* 1135 infected females (n=10) crossed with healthy males (n=5). BLI signal of the (A) testes (B) seminal vesicles of individual male offspring. BLI signal of the (C) uterii and (D) ovaries of individual female offspring.
