# Supplementary material for: Trypanosoma brucei gambiense Infections in Mice Lead to Tropism to the Reproductive Organs, and Horizontal and Vertical Transmission
Source: PLoS Negl Trop Dis. 2016 Jan 6;10(1):e0004350. doi: 10.1371/journal.pntd.0004350 (PMC4703293; doi:10.1371/journal.pntd.0004350)
Supplement: S4 Fig — 1.8% agarose gel of PCR using Tbingi-F1/pMUTec-R2 nested primers on the blood of 8 female mice taken 3.5, 5 and 6 months post-crossing with T. b. gambiense 1135(Rluc) infected male. PCR has been done in duplicate for the three series and for the two blood extracts controls. Respectively lane 1 to 11: 1-nested PCR of the first PCR negative control (water); 2-nested PCR of the first PCR positive control (1 ng T. b. gambiense DNA); 3-nested PCR of the first PCR negative control (1 ng T. congolense DNA); 4-negative control (water) of the nested PCR; 5-positive control (1 ng T. b. gambiense DNA) of the nested PCR; 6-negative control (1 ng T. congolense DNA) of the nested PCR; 7-healty mouse blood extract; 8-T. b. gambiense 1135 mouse blood extract; 9-negative control (water) of the first PCR; 10-positive control (1 ng T. b. gambiense DNA) of the first PCR; 11-negative control (1 ng T. congolense DNA) of the first PCR. Lane M: GeneRuler DNA ladder (Thermo Scientific). (DOCX) [file pntd.0004350.s004.docx]

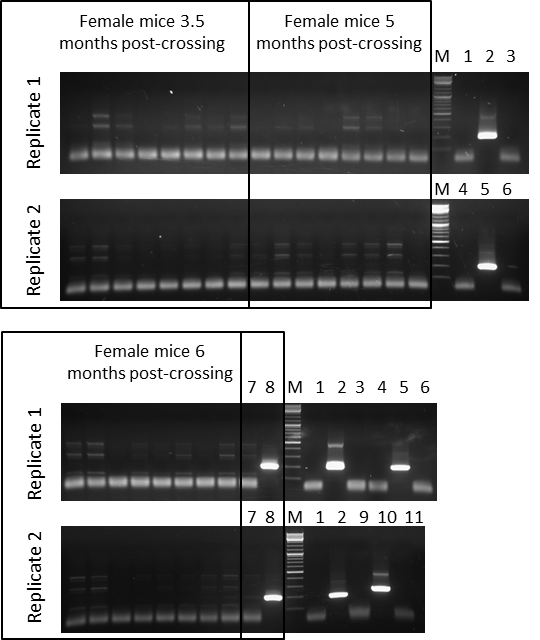


S4 Figure. Investigation of the horizontal transmission of *T. b. gambiense* 1135 (Rluc). 1.8% agarose gel of PCR using Tbingi-F1/pMUTec-R2 nested primers on the blood of 8 female mice taken 3.5, 5 and 6 months post-crossing with *T. b. gambiense* 1135(Rluc) infected male. PCR has been done in duplicate for the three series and for the two blood extracts controls. Respectively lane 1 to 11 : 1-nested PCR of the first PCR negative control (water); 2-nested PCR of the first PCR positive control (1 ng *T. b. gambiense* DNA); 3-nested PCR of the first PCR negative control (1 ng *T. congolense* DNA); 4-negative control (water) of the nested PCR; 5-positive control (1 ng *T. b. gambiense* DNA) of the nested PCR; 6-negative control (1 ng *T. congolense* DNA) of the nested PCR; 7-healty mouse blood extract; 8-T. b. gambiense 1135 mouse blood extract; 9-negative control (water) of the first PCR; 10-positive control (1 ng *T. b. gambiense* DNA) of the first PCR; 11-negative control (1 ng *T. congolense* DNA) of the first PCR. Lane M: GeneRuler™ DNA ladder (Thermo Scientific).
