# Supplementary material for: Trypanosoma brucei gambiense Infections in Mice Lead to Tropism to the Reproductive Organs, and Horizontal and Vertical Transmission
Source: PLoS Negl Trop Dis. 2016 Jan 6;10(1):e0004350. doi: 10.1371/journal.pntd.0004350 (PMC4703293; doi:10.1371/journal.pntd.0004350)
Supplement: S5 Fig — 1.8% agarose gel of PCR using Tbingi-F1/pMUTec-R2 nested primers on organs of an infected T. b. gambiense 1135 pregnant female mouse. Respectively lane 1 to 19: 1-lung; 2-intestine; 3-liver; 4-kidney; 5-placenta; 6-spleen; 7-brain; 8-ovary; 9-spinal cord; 10-uterus; 11-heart; 12-embryo; 13-embryo; 14-healthy mouse blood extract; 15-nested PCR of the first PCR negative control (water); 16-nested PCR of the first PCR positive control (1 ng T. b. gambiense DNA); 17-nested PCR of the first PCR negative control (1 ng T. congolense DNA); 18-negative control (water) of the nested PCR; 19-positive control (1 ng T. b. gambiense DNA) of the nested PCR. Lane S: other tissue samples. Lane M: GeneRuler DNA ladder (Thermo Scientific). (DOCX) [file pntd.0004350.s005.docx]

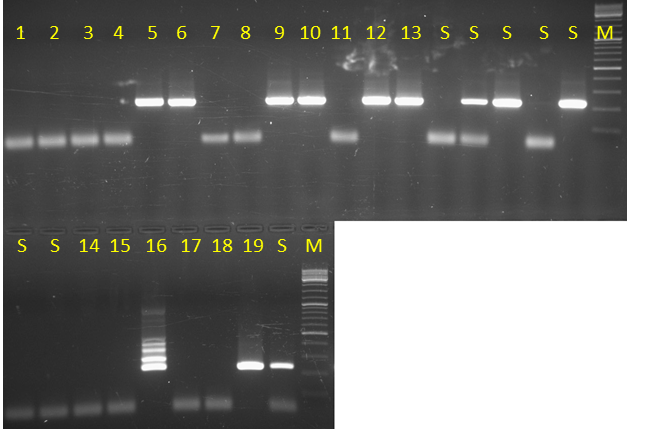


S5 Figure. Investigation of the vertical transmission of *T. b. gambiense* 1135 (Rluc). 1.8% agarose gel of PCR using Tbingi-F1/pMUTec-R2 nested primers on organs of an infected *T. b. gambiense* 1135 pregnant female mouse. Respectively lane 1 to 19 : 1-lung; 2-intestine; 3-liver; 4-kidney; 5-placenta; 6-spleen; 7-brain; 8-ovary; 9-spinal cord; 10-uterus; 11-heart; 12-embryo; 13-embryo; 14-healthy mouse blood extract; 15-nested PCR of the first PCR negative control (water); 16-nested PCR of the first PCR positive control (1 ng *T. b. gambiense* DNA); 17-nested PCR of the first PCR negative control (1 ng *T. congolense* DNA); 18-negative control (water) of the nested PCR; 19-positive control (1 ng *T. b. gambiense* DNA) of the nested PCR. Lane S: other tissue samples. Lane M: GeneRuler™ DNA ladder (Thermo Scientific).
