# Supplementary material for: Trypanosoma brucei gambiense Infections in Mice Lead to Tropism to the Reproductive Organs, and Horizontal and Vertical Transmission
Source: PLoS Negl Trop Dis. 2016 Jan 6;10(1):e0004350. doi: 10.1371/journal.pntd.0004350 (PMC4703293; doi:10.1371/journal.pntd.0004350)
Supplement: S1 Table — (DOCX) [file pntd.0004350.s006.docx]

S1 Table. Primer sequences used in the study for the sensitive and specific detection of very low parasitaemia.

| **Name** | **Primers sequence** | **Product size (bp)** | **Specificity** | **Reference** |
| --- | --- | --- | --- | --- |
| TBR1/ 2 | TBR1: GAATATTAAACAATGCGCAG | 164 | Trypanozoon | Masiga et al. (1992) [[30](#_ENREF_30)] |
|  | TBR2: CCATTTATTAGCTTTGTTGC |  |  |  |
| TBR1N/ 2 | TBR1N: CGAATGAATATTAAACAATGCGCAGT | 169 | Trypanozoon | This study |
|  | TBR2: CCATTTATTAGCTTTGTTGC |  |  |  |
| pMUTec | pMUTec F: TGCAGACGACCTGACGTACT | 227 | *T. evansi* | Wuyts et al. (1994) [[33](#_ENREF_33)] |
|  | pMUTec R: CTCCTAGAAGCTTCGGTGTCCT |  |  |  |
| pMUTec/TBingi | pMUTEc-F8 : CCAGAAGCAGAACATTTGAGCG | 241 | Trypanozoon | This study |
|  | TBingi-R1 : TGCACTTTGTTTTCGCTACG |  |  |  |
| pMUTec/TBingi nested | TBingi-F1 : AGTGCCACAAGGAACTGTCC | 201 | Trypanozoon | This study |
|  | pMUTEc-R2 : CGTGGTGTTACAGTGGTCAAAAGA |  |  |  |
