# Supplementary material for: Trypanosoma brucei gambiense Infections in Mice Lead to Tropism to the Reproductive Organs, and Horizontal and Vertical Transmission
Source: PLoS Negl Trop Dis. 2016 Jan 6;10(1):e0004350. doi: 10.1371/journal.pntd.0004350 (PMC4703293; doi:10.1371/journal.pntd.0004350)
Supplement: S2 Table — Positive samples are indicated in bolda. (DOCX) [file pntd.0004350.s007.docx]

S2 Table. Fold increase of BLI signal to control from *ex-vivo* organs of *T. b. gambiense* 1135 infected male (n=6) and female mice (n=8). Positive samples are indicated in bold^a^.

| Mouse | | **Ovaries and Uteri^b^** | **Testes** | **Seminal Vesicles** | **Brain** | **Spinal Cord** | **Spleen** | **Liver** | **Lungs** | **Kidneys** | **Intestines** |
| --- | --- | --- | --- | --- | --- | --- | --- | --- | --- | --- | --- |
| Males | 27711 SM | NA | **3.6** | **3.1** | 1.0 | **2.7** | 0.9 | 0.5 | 0.3 | **2.1** | 0.6 |
|  | 33 | NA | **2.2** | **13.2** | **1.9** | 0.8 | **2.1** | 0.5 | 0.7 | **2.2** | 1.0 |
|  | 35 | NA | 0.1 | **3.8** | 0.7 | **2.0** | 0.4 | 0.4 | 1.3 | 0.9 | 1.2 |
|  | 32 | NA | 1.2 | 1.3 | **1.7** | 1.5**^c^** | 1.7**^c^** | 0.5 | 1.0 | 1.4 | 0.7 |
|  | 28 | NA | **2.0** | **2.8** | **2.3** | 0.1 | 0.5 | 0.8 | 0.6 | 1.2 | 0.6 |
|  | 29 | NA | **4.0** | **4.2** | 0.1 | 0.1 | 0.2 | 0.2 | 0.8 | **2.7** | **2.0** |
| Females | 23365dg | **2.2** | NA | NA | 0.2 | 0.7 | 0.1 | 0.1 | 0.2 | 0.4 | 0.2 |
|  | 23365dd | 1.5**^c^** | NA | NA | 0.2 | 0.8 | 0.1 | 0.9 | 0.1 | 0.3 | 0.6 |
|  | 23795g | **2.0** | NA | NA | 0.8 | **1.9** | 0.6 | 1.0 | 0.7 | 1.2 | 0.8 |
|  | 23797g | **1.8** | NA | NA | **1.9** | **2.3** | 0.3 | 0.8 | 0.4 | 0.3 | 0.9 |
|  | 23799dd | 1.4 | NA | NA | 0.8 | 1.4 | **4.6** | 1.0 | 0.8 | 0.6 | **1.9** |
|  | 23605sm | 1.4 | NA | NA | 1.2 | 0.2 | 0.1 | 1.3 | 0.5 | 0.7 | 0.5 |
|  | 23605dd | **15.3** | NA | NA | **2.1** | **6.4** | **2.7** | 0.8 | 1.2 | **1.7** | 1.1 |
|  | 23605g | 1.2 | NA | NA | **2.3** | **12.5** | 0.7 | 1.0 | **2.3** | 1.6**^c^** | 1.3 |

^a^ Samples were considered positive if ratio of signal over the control was greater than 1.5 and absolute signal greater than the mean control intensity plus standard deviation.

^b^ Ovaries and uteri were not separated for analysis.

^c^ Samples considered negative since mean control intensity plus standard deviation was less than the control.
