# Supplementary material for: Trypanosoma brucei gambiense Infections in Mice Lead to Tropism to the Reproductive Organs, and Horizontal and Vertical Transmission
Source: PLoS Negl Trop Dis. 2016 Jan 6;10(1):e0004350. doi: 10.1371/journal.pntd.0004350 (PMC4703293; doi:10.1371/journal.pntd.0004350)
Supplement: S3 Table — Positive samples are indicated in bolda. (DOCX) [file pntd.0004350.s008.docx]

S3 Table. Investigation of sexual transmission of *T. b. gambiense* 1135 from infected male mice to healthy female mice by *ex-vivo* organ BLI and PCR. Positive samples are indicated in bold^a^.

| **Mouse** | **Ovaries** | **Uterus** | **Brain** | **Spinal Cord** | **Spleen** | **Liver** | **Lungs** | **Kidneys** | **Intestines** | **Heart** |
| --- | --- | --- | --- | --- | --- | --- | --- | --- | --- | --- |
| 229 | **2.6** | **6.1** | 0.6 | 0.6 | 1.1 | 0.9 | **2.0** | 0.8 | 1.9**^b^** | **1.6** |
| 228 | 1.0 | 0.6 | 1.1 | 0.9 | 1.2 | 0.9 | 0.8 **^b^** | 0.8 | **1.7** | 1.1 |
| 271 | **1.8** | 1.2 | 1.5^c^ | 0.7 | 1.0 | 1.2 | 2.1**^b^** | **1.8** | 1.8**^b^** | 1.2 |
| 225 | **3.9** | **1.8** | 1.3 | 0.5 | 1.2 | 0.9 | 1.6**^b^** | **1.9** | **1.8** | 1.4 |
| 275 | 0.3 | 0.6 | 1.1 | 0.6 | 1.2 | 0.7 | 0.5 | 0.9 | 0.9 | 1.2 |
| 272 | **1.8** | **1.6** | 1.0 | 0.6 | **2.3** | 0.7 | 0.6 | 1.3 | 1.2 | 1.4 |
| 220 | **1.5** | **1.9** | **1.5** | 0.4 | 0.8 | 0.7 | **2.3** | 1.3 | 1.3 | 1.4 |
| 226 | 0.3 | 0.9 | **1.8** | 0.5 | 0.7 | 0.7 | **2.4** | 0.8 | 0.7 | 1.3 |

^a^ Samples were considered positive if ratio of signal over the control was greater than 1.5 and absolute signal greater than the mean control intensity plus standard deviation.

^b^ BLI signal exterior to organ, therefore result not included in analyses.

^c^ Samples considered negative since mean control intensity plus standard deviation was less than the control.
