# Supplementary material for: Trypanosoma brucei gambiense Infections in Mice Lead to Tropism to the Reproductive Organs, and Horizontal and Vertical Transmission
Source: PLoS Negl Trop Dis. 2016 Jan 6;10(1):e0004350. doi: 10.1371/journal.pntd.0004350 (PMC4703293; doi:10.1371/journal.pntd.0004350)
Supplement: S4 Table — Positive values+ are indicated in bold a. (DOCX) [file pntd.0004350.s009.docx]

**S4 Table. Fold increase of BLI signal to control for five female and five male offspring of *T. b. gambiense* 1135 infected females (n=10) and healthy male mice (n=5). Positive values+ are indicated in bold ^a^.**

|  | **Mouse** | **Testes** | **Seminal vesicles** | **Uterus** | **Ovaries** | **Brain** | **Spinal Cord** | **Spleen** | **Liver** | **Lungs** | **Kidneys** | **Intestines** | **Heart** |
| --- | --- | --- | --- | --- | --- | --- | --- | --- | --- | --- | --- | --- | --- |
| Males | 33289 | **2.0** | 0.1 | NA | NA | 0.0 | 0.5 | 0.4 | 0.0 | 0.0 | 0.6 | 0.2 | 0.4 |
|  | 32065 | **2.1** | 0.7 | NA | NA | 0.6 | 0.7 | 0.8 | 0.2 | 0.9 | 1.4 | 0.8 | 0.3 |
|  | 32486(1) | **3.0** | **1.5** | NA | NA | 0.1 | 1.0 | 0.2 | 0.4 | 0.4 | 0.9 | 0.2 | 0.4 |
|  | 32486(2) | **2.6** | **2.2** | NA | NA | 0.8 | **3.0** | 0.5 | 0.1 | 0.0 | **1.9** | 0.8 | 0.6 |
|  | 32156 | 0.9 | 0.1 | NA | NA | 0.4 | 1.1 | 0.7 | 0.2 | 0.2 | 0.6 | 0.3 | 0.2 |
| Females | 33290 | NA | NA | 0.2 | 1.3 | 0.9 | 0.9 | 0.3 | 0.0 | 0.2 | 0.3 | 0.2 | 0.7 |
|  | 33147 | NA | NA | 1.4 | **1.9** | 0.9 | 1.5^b^ | 0.7 | 0.2 | 0.9 | 0.6 | 0.6 | 0.6 |
|  | 32925(1) | NA | NA | **2.2** | **5.9** | 1.1 | 1.5^b^ | 0.9 | 0.2 | 1.1 | 0.7 | 1.6^b^ | 0.4 |
|  | 32925(2) | NA | NA | 1.4 | **4.2** | 0.4 | 0.5 | 0.1 | 0.4 | 0.0 | 0.5 | 0.2 | 0.6 |
|  | 32925(3) | NA | NA | 1.0 | 0.2 | 0.4 | 0.2 | 0.6 | 0.1 | 0.3 | 0.4 | 0.3 | 0.3 |

NA, not applicable.

^a^ Samples were considered positive if ratio of signal over the control was greater than 1.5 and absolute signal greater than the mean control intensity plus standard deviation.

^b^ Samples considered negative since mean control intensity plus standard deviation was less than the control.
